# The effectiveness of Internet‐delivered treatment for generalized anxiety disorder: An updated systematic review and meta‐analysis

**DOI:** 10.1002/da.23115

**Published:** 2020-11-22

**Authors:** Nora Eilert, Angel Enrique, Rebecca Wogan, Olwyn Mooney, Ladislav Timulak, Derek Richards

**Affiliations:** ^1^ School of Psychology, E‐Mental Health Research Group, Trinity College Dublin University of Dublin Dublin Ireland; ^2^ Clinical Research & Innovation, SilverCloud Health Dublin Ireland

**Keywords:** anxiety, generalized anxiety disorder, Internet‐based treatment, Internet‐delivered treatment, meta‐analysis, randomized controlled trial

## Abstract

**Background:**

Generalized anxiety disorder (GAD) is a highly prevalent, chronic disorder associated with impaired quality of life, societal burden, and poor treatment rates. Internet‐delivered interventions may improve the accessibility of treatments and are increasingly being used. This study aimed to update a previous meta‐analysis to determine the effectiveness of available Internet‐delivered interventions in treating symptoms of GAD.

**Method:**

Systematic literature searches were conducted (through April 2020) using Embase, PubMed, PsychINFO, and Cochrane to find randomized controlled trials of Internet‐delivered interventions for GAD. Risk of bias was evaluated, and Hedge's *g* was calculated at posttreatment and follow‐up.

**Results:**

Twenty studies met eligibility criteria and were included in the meta‐analysis. Random‐effect models detected large effect sizes for primary outcomes of anxiety (*g* = 0.79) and worry (*g* = 0.75), favoring treatment. Effect sizes for depression, functional impairment, and quality of life were moderate to large. Maintenance of effects at follow‐up seems likely.

**Conclusions:**

Results support the effectiveness of Internet‐delivered treatments for GAD. Considerable heterogeneity between studies appeared moderated by variability in the interventions themselves, highlighting the importance of further investigation into the characteristics that may optimize treatment outcomes. Overall, Internet‐delivery appears to be a viable mode of treatment for GAD with potential to relieve existing gaps in the provision of treatment.

## 1 INTRODUCTION

Generalized anxiety disorder (GAD) is a disorder characterized by excessive anxiety and worry, as well as associated symptoms such as restlessness, fatigue, irritability, muscle tension, and/or sleep disturbance, which for a diagnosis to be made, need to have been present more days than not for at least 6 months (American Psychiatric Association, 2013). Over the course of a life‐time, GAD tends to have a chronic nature. It is one of the most prevalent anxiety disorders, with lifetime prevalence estimated internationally at approximately 6% (Bandelow & Michaelis, 2015; Remes et al., 2016). GAD is significantly and negatively associated with personal and health‐related quality of life (Olatunji et al., 2007; Saarni et al., 2007), appears to also have a significant societal impact (Wittchen, 2002), and is a highly comorbid disorder (Brown et al., 2001).

There are currently several forms of treatment recommended for GAD, including some form of antidepressant medication (Strawn et al., 2018). In terms of psychotherapy, several forms of therapy, particularly variations of cognitive‐behavioral therapies (CBTs) have been empirically supported (Behar et al., 2009). These therapies offer a variety of therapeutic strategies targeting beliefs around worry and emotional avoidance as present in patients with GAD. More recently, CBT transdiagnostic approaches have also been applied to patients with GAD (Barlow et al., 2017). In the context of calls to establish evidence for non‐CBT models (Hunot et al., 2007) and to increase patients' choice of treatment (Williams et al., 2016), noncognitive behavioral models have also emerged and are being evaluated (e.g., Keefe et al., 2014).

An important factor in the delivery of treatments for GAD in large public health services is the so‐called “stepped care,” which addresses issues of availability and cost‐effectiveness, by offering interventions on a continuum from low‐to‐high intensity. For instance, in the National Health Service in the United Kingdom, stepped‐care provision for the treatment of GAD (National Institute for Health and Care Excellence, 2011) recommends for less distressed patients guided self‐help and psychoeducation in a group format based on the principles of CBT. Similarly, collaborative care models advocate for the integration of behavioral and general medical services in primary care and the delivery of a range of evidence‐based low and high intensity interventions to patients to achieve specific clinical goals (Vanderlip et al., 2016). An alternate form of low‐intensity intervention, which may fit into stepped and collaborative care models, is Internet‐delivered treatment, often in the form of Internet‐delivered cognitive behavioral therapy (iCBT). This form of treatment was developed in recent decades for a variety of mental health conditions, particularly depression and anxiety disorders including GAD (e.g., Arnberg et al., 2014).

Given that GAD is one of the most highly prevalent anxiety disorders, of a very chronic nature, which often goes untreated (Dell′Osso et al., 2013), and given that Internet‐delivered treatments offer the possibility of fast dissemination of available treatments, we wanted to examine the effectiveness of available Internet‐delivered GAD treatments. Specifically, we aimed to systematically review all evidence for Internet‐delivered interventions targeting GAD and thereby update a systematic review and meta‐analysis conducted by Richards et al. (2015). While there are several recent meta‐analyses of psychological treatments of GAD, some of which included Internet‐delivered interventions (e.g., Carl et al., 2020; Chen et al., 2019; Hall et al., 2016), none specifically set out to evaluate all available evidence for Internet‐delivered interventions (e.g., they only included iCBT, focused only on specific subgroups like older adults, or Internet‐delivered interventions were included alongside other self‐help and face‐to‐face interventions).

As Internet‐delivered treatments are developing fast and we have been aware of a number of new developments in the area of Internet‐delivered treatments for GAD (e.g., Dahlin et al., 2016; Richards et al., 2016), we wanted to build on the work of Richards et al. (2015) and provide a fresh look at the available treatment options and their effectiveness. In doing so, we also strived to offer an in‐depth look at some moderators that may influence the effectiveness of Internet‐delivered treatments for GAD; for example, type of control group used (Zhu et al., 2014), specific intervention characteristics like theoretical orientation, composition or length of the treatment (Zhang et al., 2019), sample characteristics (Carl et al., 2020), and support offered (Wright et al., 2019). There were therefore two questions that guided our review (Eilert et al., 2019): Are Internet‐delivered treatments effective in treating symptoms of GAD when compared to control conditions? What are the moderators of the effectiveness of Internet‐delivered interventions in treating symptoms of GAD?

## 2 METHODS

### 2.1 Literature search

This systematic review and meta‐analysis was conducted according to the PRISMA statement (Moher et al., 2009; for the corresponding checklist see Appendix A). A systematic literature search for English language articles was conducted across four prominent electronic databases: Embase, PubMed, PsychINFO, and Cochrane. The search was carried out in two stages, the first one in December 2018 and a more updated one in April 2020. Only publications published after June 1, 2013 (cut‐off for articles included in original review) were included in the searches. To facilitate an update of the previous meta‐analysis, we used the same two key search phrases “Internet treatment for generalized anxiety disorder” and “Internet treatment for anxiety.” We used the same two search phrases across the four databases to replicate procedures in the original meta‐analysis. This resulted in a total of eight searches. In addition, where protocols meeting eligibility criteria were identified through the searches, we looked up the initial protocol registration on the corresponding trial register for recent publications associated with it. We also cross‐checked references of other relevant review papers.

### 2.2 Selection of studies

Following the removal of duplicates, initial search results were screened at title/abstract by one researcher (N. E. Dec’18’ searches/R. W. April’20’ searches) and entirely off‐topic studies were excluded. Remaining papers were assessed for eligibility by reading full texts and grounds for exclusion were recorded in line with a predefined hierarchy. Eligibility assessments were carried out independently by two researchers (N. E. and A. E. Dec’18’ searches/R. W. and N. E. April’20’ searches) and discrepancies in study selection that arose were solved through discussion and consultation with a senior researcher (D. R.).

Eligibility criteria were established to solely include studies that were (1) an Internet‐delivered intervention for symptoms of GAD; (2) a randomized controlled trial (RCT); (3) compared with a wait‐list, placebo, or attention control; (4) had a sample with a confirmed clinical diagnosis of GAD who may have had comorbidities and/or impairment in functioning (studies that included a minority of participants with significant subthreshold symptoms of GAD were also accepted); (5) had a sample of adults over 18 years of age; (6) published in a peer‐reviewed journal in English and; (7) included reliable and valid outcome measures of symptoms of GAD (i.e., measures of anxiety and/or worry).

In addition, for transdiagnostic protocols and studies reporting on outcomes across several anxiety disorders in total only, we contacted authors to request the discrete outcome data pertaining to participants with a confirmed diagnosis of GAD. Where authors were unable to provide the required data, studies were excluded from the meta‐analysis.

### 2.3 Data extraction

We extracted the following data from included studies: (a) country where study was conducted; (b) participant/sample characteristics (diagnostic measure used, mean age, baseline symptom levels, method of referral); (c) scope of intervention (transdiagnostic or disorder‐specific); (d) intervention type (i.e., iCBT, Internet‐delivered psychodynamic therapy etc.); (e) type of control group; (f) support during intervention; (g) length of treatment phase and number of modules in intervention; (h) engagement metrics (percentage of program completed, average supporter time spend per participant); (i) means and standard deviation to calculate posttreatment and follow‐up effect sizes where relevant.

As some trials used numerous instruments to measure outcomes related to our five constructs of interest (primary outcomes: anxiety and worry; secondary outcomes: depression, impaired functioning, and quality of life), a hierarchy of instruments was developed before data analysis as per recommendations by the Cochrane Handbook and to facilitate uniformity where possible (B. Johnston et al., 2019). Within each construct, a list of relevant outcome measures was generated, which were then ranked based on reliability, validity, and interpretability (i.e., given our primary aim of evaluating intervention effects on GAD symptoms, anxiety measures relating more specifically to GAD like the Generalized Anxiety Questionnaire‐7 item were rated higher then generic anxiety measures like the Beck Anxiety Inventory). See Table 1 (columns 9–13) for the specifics of which outcome measure informed which construct in each study. To evaluate potential effects of using different outcome measures within each construct, the specific measure used by each study was coded to be evaluated through sensitivity analyses. We contacted the authors of 13 studies, from the original and updated searches, to extract either outcome data pertaining to GAD participants (as described above) and/or additional data relating to potential moderators of effects. Data were extracted by one researcher (N. E.) and checked for accuracy by a second one (R. W.).

**Table 1:** Characteristics of studies included in the meta‐analysis

Study (year)	Participants	Mean age (range)	Intervention (*n*)	Control (*n*)	Intervention duration weeks/number of modules	Country	Support	Outcomes				
								Anxiety	Depression	Impaired functioning	Quality of life	Worry
G. Andersson et al. (2012)	General population, self‐referral, telephone‐administered SCID‐I diagnosis	44.4 (22–66)	Multi‐arm trial: Disorder‐specific iCBT (27)	Wait‐list (27)	8/8	Sweden	iCBT: CBT trained therapists (two licensed psychologists and three final year psychology students)	GAD‐IV	BDI‐II	‐	QOLI	PSWQ
			Disorder‐specific iPDT (27)				iPDT: Psychodynamically trained therapists (three final year clinical psychology students, one licensed psychologist)					
E. Andersson et al. (2017)^a^	General population, self‐referral, telephone‐administered MINI diagnosis	32.5 (18+)	Transdiagnostic IbET (70)	Wait‐list (70)	10/8	Sweden	Therapists (final year clinical psychology students and licensed psychologists)	HAD‐A	MADRS‐S	‐	BBQ	PSWQ
Bell et al. (2012)	Secondary care service users, recruited from service wait‐list, in‐person‐administered SCID‐I diagnosis	33.6 (18–65)	Disorder‐specific cCBT (6)	Wait‐list (7)	12/4	New Zealand	Research assistants	GADI	BDI‐II	WSAS	‐	PSWQ
Berger et al. (2014)	General population, self‐referral, telephone‐administered SCID‐I diagnosis	35.1 (18–65)	Multi‐arm trial: Transdiagnostic tailored Internet‐delivered guided self‐help (10)	Wait‐list (12)	8/8	Switzerland, Germany, Austria	Minimal therapist guidance (five final year clinical psychology students, one clinical psychologist, one CBT therapist)	BAI	BDI‐II	‐	‐	PSWQ
			Standard disorder‐specific Internet‐delivered guided self‐help (11)									
Christensen et al. (2014)	General population, self‐referral, telephone‐administered MINI diagnosis	25.5 (18–30)	Disorder‐specific online e‐health iCBT (8)	Online attention placebo (7)	10/10	Australia	Clinical psychologists and GPs	GAD‐7	CES‐D	‐	‐	‐
Dahlin et al. (2016)	General population, self‐referral, telephone‐administered SCID‐I diagnosis	39.48 (18+)	Disorder‐specific Internet‐delivered acceptance‐based behavior therapy (52)	Wait‐list (51)	9/7	Sweden	Clinical psychologists	GAD‐7	PHQ‐9	‐	QOLI	PSWQ
Dear et al. (2015)	Older general population, self‐referral, telephone‐administered MINI diagnosis	65.45 (61–81)	Transdiagnostic anxiety and stress‐directed iCBT (16)	Wait‐list (23)	8/5	Australia	Clinical psychologist	GAD‐7	PHQ‐9	‐	EQ‐5D‐5L	‐
Hirsch et al. (2018)	General population, self‐referral, telephone‐administered SCID‐I diagnosis	30.59 (18–65)	Multi‐arm trial: Transdiagnostic iCBM‐I (44)	Placebo iCBM‐I (20)	4/10	The United Kingdom	Researchers	GAD‐7	PHQ‐9	‐	‐	PSWQ
Johansson et al. (2013)	General population, self‐referral, telephone‐administered MINI diagnosis	44.9 (19–77)	Transdiagnostic Internet ‐delivered APT (23)	Therapist supported wait‐list (26)	10/8	Sweden	Therapists (final year clinical psychology students)	GAD‐7	PHQ‐9	‐	‐	‐
L. Johnston et al. (2011)	General population, self‐referral, telephone‐administered MINI diagnosis	41.62 (19–79)	Multi‐arm trial: Transdiagnostic clinician‐assisted iCBT (21)	Wait‐list (20)	10/8	Australia	Clinician: clinical psychologist	GAD‐7	PHQ‐9	SDS	‐	PSWQ
			Coach‐assisted iCBT (18)				Coach: registered psychologist without specialist training					
Jones et al. (2016)	Older general population, self‐referral, telephone‐administered MINI diagnosis	65.15 (60+)	Transdiagnostic iCBT (24)	Wait‐list (22)	7–10/7	Canada	Therapist	GAD‐7	PHQ‐9	‐	WHOQOL‐BREF	PSWQ‐A
Mullin et al. (2015)	University students, self‐referral, telephone‐administered MINI diagnosis	27.7 (19–55)	Transdiagnostic iCBT (27)	Wait‐list (13)	5–6/3–6	Australia	Therapist (clinical psychology doctoral student)	GAD‐7	PHQ‐9	SDS	‐	‐
Newby et al. (2013)^a^	Wait‐list service users, self‐referral, telephone‐administered MINI diagnosis	44.25 (18+)	Transdiagnostic iCBT (46)	Wait‐list (54)	10/6	Australia	Clinician and therapist	GAD‐7	PHQ‐9	WHODAS‐II	‐	PSWQ
Paxling et al. (2011)	General population, self‐referral, telephone‐administered SCID‐I diagnosis	39.3 (18–66)	Transdiagnostic iCBT (44)	Wait‐list (45)	8/8	Sweden	Therapists (final year psychology students and clinician)	GAD‐IV	MADRS‐S	‐	QOLI	PSWQ
Richards et al. (2020)	Stepped care service users, routine care referral, PWP‐administered MINI diagnosis	33 (18–80)	Transdiagnostic iCBT (134)	Wait‐list (65)	8/7	The United Kingdom	PWPs (psychology graduates trained in CBT)	GAD‐7	PHQ‐9	WSAS	EQ‐5D‐5L	‐
Robinson et al. (2010)	General population, self‐referral, telephone‐administered MINI diagnosis	46.96 (18–80)	Multi‐arm trial: Disorder‐specific iCBT clinician‐assisted (50)	Wait‐list (48)	10/6	Australia	Clinician: clinical psychologist. Technician: clinical manager administrative role at the clinic	GAD‐7	PHQ‐9	SDS	‐	PSWQ
			Technician‐assisted (47)									
Salemink et al. (2014)	Secondary care service users, recruited from service wait‐list, telephone‐administered SCID‐I diagnosis	40.25 (18+)	Transdiagnostic iCBM‐I (11)	Placebo iCBM‐I (7)	1.6/8	The Netherlands	Unsupported	STAI‐T	BDI	‐	‐	‐
Titov et al. (2010)	General population, self‐referral, telephone‐administered MINI diagnosis	39.5 (18–74)	Transdiagnostic iCBT (18)	Wait‐list (16)	8/6	Australia	Clinical psychologists	GAD‐7	PHQ‐9	SDS	‐	PSWQ
Titov et al. (2011)	General population, self‐referral, telephone‐administered MINI diagnosis	43.9 (18–79)	Transdiagnostic iCBT (9)	Wait‐list (12)	10/8	Australia	Clinical psychologist	GAD‐7	PHQ‐9	SDS	‐	PSWQ
Titov et al. (2009)	General population, self‐referral, telephone‐administered MINI diagnosis	44 (18+)	Disorder‐specific iCBT (24)	Wait‐list (21)	9/6	Australia	Clinical psychologist	GAD‐7	PHQ‐9	SDS	‐	PSWQ

Abbreviations: APT, affect‐phobia therapy; BAI, Beck Anxiety Inventory; BBQ, Brunnsviken Brief Quality of Life Questionnaire; BDI‐II, Beck Depression Inventory‐II; cCBT, computerized cognitive behavioral therapy; CES‐D, Centre for Epidemiological Studies Depression Scale; EQ‐5D‐5L, EuroQol–5 Dimensions–5 Levels; GAD‐IV, Generalized Anxiety Questionnaire‐IV; GAD‐7, Generalized Anxiety Questionnaire‐7 item; HAD‐A, Hospital Anxiety and Depression Scale‐Anxiety; HAD‐D, Hospital Anxiety and Depression Scale‐Depression; IbET, Internet‐based extinction therapy; iCBM‐I, Internet‐based cognitive bias modification for interpretations; iCBT, Internet‐delivered cognitive behavioral therapy; iPDT, Internet‐delivered psychodynamic therapy; MADRS‐S, Montgomery‐Asberg Depression Scale; MINI, Mini International Neuropsychiatric Interview; PHQ‐9, Patient Health Questionnaire–9 item; PSWQ, Penn State Worry Questionnaire; PSQW‐A, Penn State Worry Questionnaire‐Abbreviated; PWP, psychological well‐being practitioners; QOLI, Quality of Life Inventory; SCID‐I, Structured Clinical Interview for DSM‐4 Axis I Disorders; SDS, Sheehan Disability Scale; STAI‐T, State‐Trait Anxiety Inventory‐Trait; WHODAS‐II, World Health Organization disability assessment schedule II; WHOQOL‐BREF, World Health Organization Quality of Life Instrument; WSAS, Work and Social Adjustment Scale.

^a^ These two studies included a minority of participants with significant subthreshold symptoms of GAD.

### 2.4 Quality assessment of included studies

To determine the risk of bias of each study we employed the CLEAR NPT checklist (Boutron et al., 2005), designed to measure the quality of RCTs evaluating nonpharmacological treatments (NPTs). The protocol for the meta‐analysis intended to use the Revised Cochrane Risk‐of‐Bias tool (RoB 2); however, as the investigators became aware of issues raised in relation the RoB 2 (i.e., complexity of the tool, relatively low inter‐rater reliability; Minozzi et al., 2020), along with the fact that the tool is still in the validation phase, it was decided to use another well‐established, standard for assessing the quality of RCTs. The CLEAR NPT checklist has been successfully used in previous meta‐analytic studies of Internet‐delivered interventions for depression (Wright et al., 2019). This checklist assesses studies on 10 key questions and 5 subquestions; most of which require an answer of yes/no/unclear based on whether or not the study meets the criteria for that item. The items focus on the adequacy of randomization, the accessibility of the details of the interventions, appropriacy of supporter skills, measurement of treatment adherence, whether there was blinding of everyone involved or, if not, the acknowledgment of the steps taken to prevent bias, uniformity across follow‐up schedules of conditions, and whether an intention‐to‐treat principle of analysis was followed. The CLEAR NPT checklist was independently completed for all studies by two researchers (R. W. and O. M.) in relation to our primary outcomes (anxiety and/or worry). Conflicts were discussed and resolved in consultation with NE. As the CLEAR NPT checklist does not provide an overall study quality rating, risk of bias assessment results were used to inform evaluations of the state of the body of literature as a whole rather than incorporated in meta‐analytic models on an individual basis.

### 2.5 Meta‐analytic procedures

All analyses, including the calculation of effect sizes, were conducted in R using the “metafor” and “dmeta” packages (Harrer et al., 2019; Viechtbauer, 2010). To assess posttreatment standardized mean differences between treatment and control groups, Hedge's *g* was calculated for each construct addressed within each study. In three‐arm trials in which both active arms met inclusion criteria, control group sample sizes were halved to allow for the calculation of separate effect sizes by trial arm (Higgins et al., 2019). For the evaluation of effect sizes, we implemented the following cut‐off points; 0–0.32 for a small effect, 0.33–0.55 for a moderate effect, and 0.56–1.2 was considered a large effect (Lipsey & Wilson, 1993).

Due to the anticipated moderate‐to‐high level of between‐study heterogeneity and in line with the PROSPERO protocol, random‐effect models were used to pool effect sizes. Restricted maximum likelihood (REML) was used to estimate between study variance and heterogeneity was assessed through the Q‐value, *I* ^2^ statistic and prediction/credibility intervals. According to Higgins and Thompson (2002), an *I* ^2^ value of 0% indicates no heterogeneity, 25% indicates low heterogeneity, while 50% and 75% indicate moderate and high levels, respectively. Model fit and the presence of outliers were assessed through diagnostic plots and statistics.

Follow‐up between‐group effects were assessed through the same models as posttreatment effects where this was feasible (i.e., a sufficient number of studies included relevant data). Where there were multiple follow‐up time points available for one study, the follow‐up time point closest to the ones used by other comparisons within an analysis was selected to ensure as much coherency as possible (i.e., where all but one study included only follow‐up time points under 6 months, the 6‐month rather than the 12‐month follow‐up point was selected). Two sets of sensitivity analyses were conducted to assess the robustness of conclusions drawn from the analysis. To assess the influence of studies that may potentially act as confounders within the meta‐analysis (i.e., studies where not all participants had GAD diagnoses but subthreshold symptoms) random‐effect models with and without these studies were compared. To assess the influence of potentially debatable methodological decisions (i.e., how outcome measures within constructs were selected in line with a predefined hierarchy) mixed‐effect models controlling for those decisions (i.e., by including outcome measure used within each study as a predictor of effects) were conducted.

To explore various potential moderators, mixed‐effect models of primary outcomes (anxiety and worry) utilizing the Knapp–Hartung method to reduce the chance of type 1 error were used. As per Fu et al. (2011) recommendations and as an absolute lower limit, moderators were only explored statistically if there were at least six moderate‐to‐large studies with data available for any continuous moderator and four moderate‐to‐large studies per subgroup for categorical moderators. In terms of baseline symptom severity, relative symptom severity was calculated by subtracting average from observed baseline scores across studies reporting on a specific outcome measure (Chaimani, 2015). Publication bias was examined using funnel plots and Egger et al. (1997) test was used to evaluate possible asymmetry in the latter.

## 3 RESULTS

### 3.1 Selection and inclusion of studies

**Figure 1:**
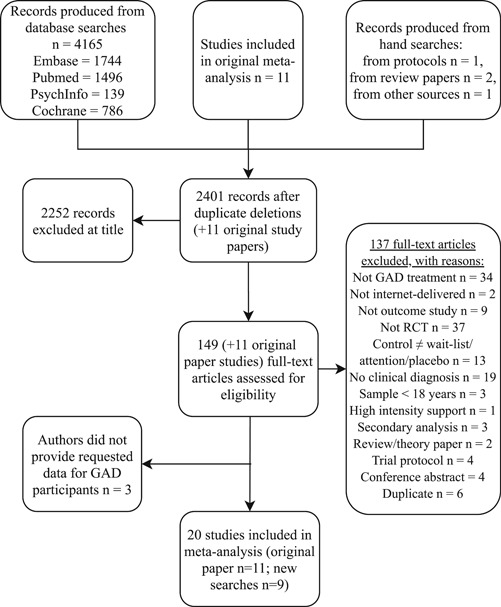
Flowchart of study inclusion and exclusion. GAD, generalized anxiety disorder; RCT, randomized controlled trial

The electronic database searches resulted in 4165 records. Four records were identified through other sources. After duplicate removal 2401 records remained. Of these, 2252 records were excluded after reviewing title and abstract, leaving a total of 149 potentially eligible records. Full‐text versions of these articles were obtained and examined for eligibility. Of those considered eligible, it was necessary to exclude three transdiagnostic studies as the authors did not provide requested data for GAD participants. Finally, nine RCTs fulfilled all eligibility criteria and were included. In addition, 11 articles were carried over from the original meta‐analysis (Richards et al., 2015), bringing to 20 the number of studies included for analysis. Figure 1 shows the results of the systematic searches, the flow of exclusions as well as the reasons for exclusion.

### 3.2 Description of included studies

The main characteristics of the included studies are presented in Table 1. Across 20 studies a total of 1333 participants were included—767 in treatment conditions and 566 control conditions. Sample size ranged from 13 (Bell et al., 2012) to 199 (Richards et al., 2020) with a mean sample size of 52. All participants were adults, ranging in age from 18 to 81. Participants across 17 studies self‐referred through websites, radio, newspapers, or email. The remaining three studies included service users from routine care settings, both primary and secondary. Five of the studies were conducted in Sweden, nine in Australia, two in the United Kingdom, one each in New Zealand, Canada, and the Netherlands, and one was conducted between Switzerland, Germany, and Austria.

All studies employed either the Structured Clinical Interview for DSM‐IV Axis I Disorders (SCID‐I; First, 1997), or the Mini International Neuropsychiatric Interview Version 5.0.0 (MINI; Sheehan et al., 1998) to establish a clinical diagnosis. Most studies administered these interviews over the telephone, while one was conducted in person. Within one study, 76% of the sample fulfilled a diagnosis of GAD but the remainder of participants had significant subthreshold symptoms (E. Andersson et al., 2017). Newby et al. (2013) also included participants with subthreshold GAD and a diagnosis of major depressive disorder (MDD; 15% of the sample). Sensitivity analyses were conducted to ensure the small numbers of participants without a full GAD diagnosis across these studies did not unduly influence meta‐analytic outcomes.

In 18 comparisons, regular support was provided by a qualified or soon‐to‐be qualified therapist or clinician. Five received support in the form of psychological well‐being practitioners, nonspecialist psychologists, technicians, or researchers, and another study was unsupported. Support was mainly offered with a view to reinforcing engagement, normalizing difficulties, and giving guidance throughout the program completion. Support consisted of individual feedback, answering questions, and feedback on assignments. This was predominantly given through email or telephone, but also through text messaging, webpage messaging, video conferencing, and online discussion forums. Within two studies comparing supporter types (L. Johnston et al., 2011; Robinson et al., 2010), clinician supporters were also allowed to provide clients with information about further skill implementation and development of strategies and goals. Seventeen studies were wait‐list‐controlled while four implemented active control groups (one attention placebo health website, one therapist‐supported attention group, two placebo cognitive bias modification programs). The duration of treatment periods ranged from 1.6 to 12 weeks.

### 3.3 Program completion and support utilization

For each study, the average percentage of the intervention program completed by participants was estimated within four categories (0%–25%, 26%–50%, 51%–75%, 76%–100%) as some articles did not allow for the calculation of exact percentages. Program completion was addressed in 19 out of the 20 studies. One study did not include measures of program completion (Christensen et al., 2014). For most interventions (*n* = 15), the researchers reported an average amount of program completion between 76%–100%, six reported between 51% and 75%, and one study reported a percentage completion in the range of 26%–50%. The average amount of time supporters spent per person over the course of their treatment was 77.04 min across the 15 studies providing this information, ranging from 18.15 min (Newby et al., 2013) to 130 min (Titov et al., 2009).

### 3.4 Intervention characteristics

Overall, nine interventions were disorder‐specific and 15 were transdiagnostic. A total of 18 interventions were based on conventional (second wave) CBT (seven of which were disorder‐specific), two on cognitive bias modification, one on psychodynamic therapy (disorder‐specific), one on extinction therapy, one on acceptance‐based behavior therapy (disorder‐specific), and one on affect‐focused psychodynamic psychotherapy. Most interventions consisted of eight modules (i.e., units of content delivered to participants). Specifically, there were 12 interventions with 8 modules, 4 with 6 modules, 2 with 10 modules, 3 with 7 modules, 1 with 5 modules, 1 with 4 modules, and 1 with a range of 3–6 modules.

With the exception of the two interventions based on cognitive bias modification, most interventions included some combination of the following nonspecific intervention components: psychoeducation, case examples or vignettes, mindfulness and/or relaxation exercises, notification and/or reminder emails, homework, summaries, and relapse prevention and maintenance. In some interventions, participants had to have completed homework assignments or modules to progress to the next module. Several interventions included additional, supplementary information and resources on lifestyle factors, for example, sleep, interpersonal relationships, communication, problem‐solving, and stress.

Given differences in the models used across interventions, there was variability between iCBT and other approaches in terms of the specific techniques used. In iCBT interventions, specific cognitive techniques included cognitive restructuring, cognitive distancing, thought and worry records or diaries, mood monitoring; and behavioral techniques included activity scheduling, behavioral experiments, graded exposure, worry exposure, scheduled worry time; and homework. Specific techniques used in other interventions included defense restructuring, extinction techniques, functional analysis, valued action, acceptance, affect‐experiencing techniques, affect expression, and cognitive bias modification. Intervention components that specifically addressed worry, like psychoeducational content, for example, types of worry, and worry‐related techniques, for example, cognitive restructuring of worry‐related beliefs, were reported in 15 interventions.

### 3.5 Quality of studies

Based on the criteria outlined in the CLEAR NPT checklist guidelines, overall, methodological quality assessment ratings seemed good across included studies with 90% (18/20) or more reporting an adequate method of allocation sequence generation, providing clear descriptions of the intervention administered, quantitatively assessing participant adherence, and analyzing outcomes in line with the intention‐to‐treat principle. The percentage of studies that reported an adequate method of allocation concealment and appropriate level of care provider experience or skill was slightly less, at 75% (15/20) and 80% (16/20), respectively. Given the nature of nonpharmacological trials and self‐report outcome assessment, adequate blinding of participants, care providers, and outcome assessors to treatment allocation was often not feasible in the included studies. Related checklist items were, therefore, among the most poorly rated with participants and outcome assessors blinded in only 10% (2/20) of studies and care providers blinded in only 5% (1/20) of studies.

**Figure B1:**
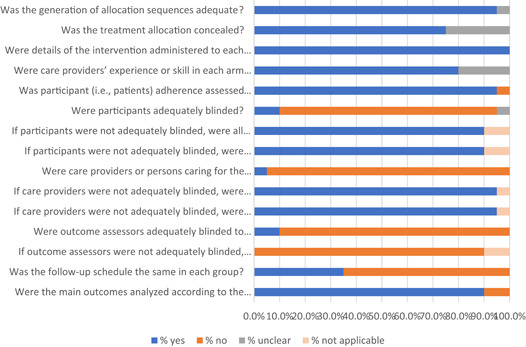
Quality ratings from risk of bias assessment for included studies

However, in studies where participants and care providers were not blinded, the provision of all other treatments and care, and the number of withdrawals and loss to follow‐up were the same in each randomized group, which may have helped to minimize the risk of bias associated with lack of blinding. In studies where outcome assessors were not blinded, no study reported on specific methods used to avoid ascertainment bias (i.e., systematic differences in outcome assessment). Only 35% (7/20) of studies adhered to the same follow‐up schedule for randomized groups with discrepancies often related to shorter follow‐up schedules for waiting list groups, possibly due to ethical considerations surrounding the withholding of treatment. Quality ratings for included studies are displayed in Figure B1 (see Appendix B).

### 3.6 Meta‐analysis of primary and secondary outcomes

#### 3.6.1 Random‐effect model for anxiety

**Figure 2:**
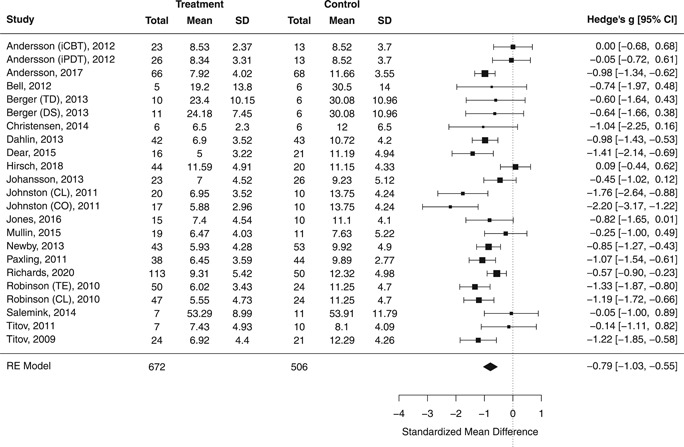
Posttreatment standardized mean difference between Internet‐delivered treatment and control group for anxiety outcomes. CL, clinician‐supported; CO, coach‐supported; DS, disorder‐specific intervention; iCBT, Internet‐delivered cognitive behavioral therapy; iPDT, Internet‐delivered psychodynamic therapy; TD, transdiagnostic intervention; TE, technician‐supported

Based on 23 comparisons (across 19 studies), a large effect on symptoms of anxiety in favor of the Internet‐delivered treatment was detected (*g* = −0.79; 95% confidence interval [CI], −1.03, −0.55; *p* < .0001; see Figure 2). However, heterogeneity, so the variability in the observed effect sizes, appeared moderate to high (*Q*_(22)_ = 56.76; *p* < .0001; *I* ^2^ = 64.99%), with the wide prediction interval (PI) crossing the zero line of no effect (95% PI, −1.69, 0.11). Model diagnostics suggested two potential outliers (Hirsh et al., 2018; L. Johnston et al., 2011—coach support arm). Excluding these two studies from the analysis resulted in the reduction of heterogeneity but a comparable effect estimate (*g* = −0.80; −1.0 −0.61; *p* < .0001; *Q*_(20)_ = 37.89; *p* = .009; *I* ^2^ = 49.89%) and narrower PI not crossing zero anymore (95% PI, −1.47, −0.13).

#### 3.6.2 Random‐effect model for worry

**Figure 3:**
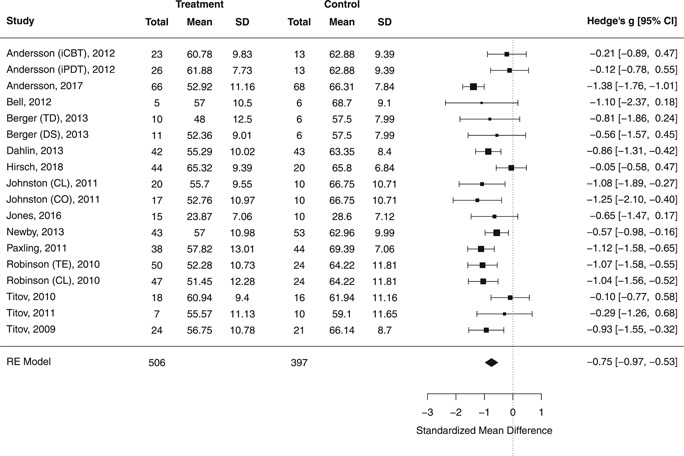
Posttreatment standardized mean difference between Internet‐delivered treatment and control group for worry outcomes. CL, clinician‐supported; CO, coach‐supported; DS, disorder‐specific intervention; iCBT, Internet‐delivered cognitive behavioral therapy; iPDT, Internet‐delivered psychodynamic therapy; TD, transdiagnostic intervention; TE, technician‐supported

For worry, outcomes across 18 comparisons (14 studies) suggested a large effect favoring the treatment (*g* = −0.75; 95% CI, −0.97, −0.53; *p* < .0001; see Figure 3). Heterogeneity was moderate (*Q*_(17)_ = 36.22; *p* = .004; *I* ^2^ = 53.38%) and the PI ranged from −1.48 to −0.02. One potential outlier was detected (E. Andersson et al., 2017), whose removal resulted in a similar effect estimate but improved heterogeneity metrics (*g* = −0.69; 95% CI, −0.90, −0.45; *p* < .0001; *Q*_(16)_ = 25.51; *p* = .061; *I* ^2^ = 40.80%; 95% PI, −1.30, −0.09).

#### 3.6.3 Random‐effect model for depression

**Figure B2:**
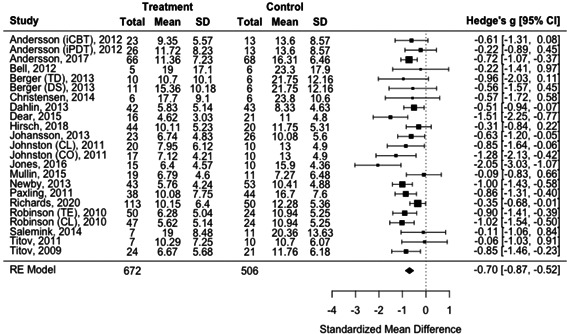
Posttreatment standardized mean difference between Internet‐delivered treatment and control group for depression outcomes. CL, clinician‐assisted; CO, coach‐assisted; DS, disorder‐specific; iCBT; Internet‐delivered cognitive behavioral therapy; iPDT, Internet‐delivered psychodynamic therapy; RE, random effect; TD, transdiagnostic; TE, technician‐assisted

Drawing on 23 comparisons (across 19 studies) for depressive symptoms, a large effect of treatment was observed (*g* = −0.70; 95% CI, −0.87, −0.52; *p* < .0001; see Appendix B, Figure B2). Heterogeneity was found to be low but significant (*Q*_(22)_ = 34.35; *p* = .045; *I* ^2^ = 31.24%) with the PI reaching from −1.16 to −0.24.

#### 3.6.4 Random‐effect model for functional impairment

**Figure B3:**
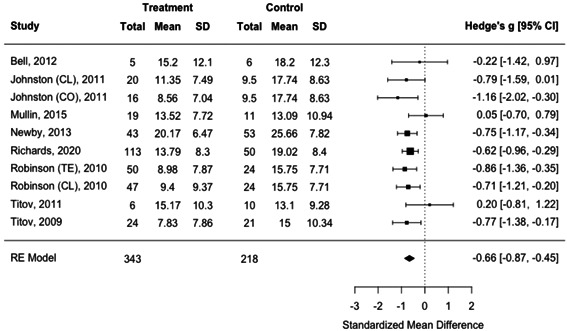
Posttreatment standardized mean difference between Internet‐delivered treatment and control group for impaired functioning outcomes. CL, clinician‐assisted; CO, coach‐assisted; RE, random effect; TE, technician‐assisted

The overall effect of treatment on levels of functioning across 10 comparisons (eight studies) was also large (*g* = −0.66; 95% CI, −0.87, −0.45; *p* < .0001; see Appendix B, Figure B3). The *Q* test for heterogeneity was nonsignificant (*Q*_(9)_ = 9.1326; *p* = .42), *I* ^2^ was 0.00% (95% CI, 0%, 83.54%) and the PI was rather narrow (95% PI, −0.87, −0.45).

#### 3.6.5 Random‐effect model for quality of life

**Figure B4:**
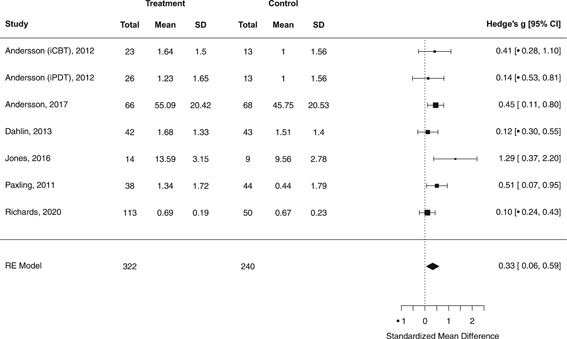
Posttreatment standardized mean difference between Internet‐delivered treatment and control group for quality of life outcomes. iCBT; Internet‐delivered cognitive behavioral therapy; iPDT, Internet‐delivered psychodynamic therapy; RE, random effect

In terms of quality of life, outcomes across seven comparisons (six studies) suggested a small‐to‐moderate effect in favor of the treatment (*g* = 0.33; 95% CI, 0.06, 0.59; *p* = .024; see Appendix B, Figure B4). While the *I* ^2^ and *Q* value metric suggested little heterogeneity (*Q*_(6)_ = 8.44; *p* = .21; *I* ^2^ = 15.70%), the *I* ^2^ 95% CI [0%, 92.05%] and the 95% PI was rather wide [−0.04, 0.70], suggesting some caution around the reliability of this result nonetheless.

### 3.7 Meta‐analysis of follow‐up outcomes

Six of the studies included follow‐up assessments of treatment and control groups (G. Andersson et al., 2012; Bell et al., 2012; Christensen et al., 2014; Hirsch et al., 2018; Salemink et al., 2014; Titov et al., 2010), rendering only the pooling of effect size across anxiety, depression, and worry outcomes feasible. Including six comparisons (five studies; treatment: *n* = 98, control: *n* = 59) with follow‐up periods ranging from 1‐ to 6‐months, follow‐up anxiety effect estimates were found to be large (*g* = −0.84; 95% CI −1.21, −0.46; *p* = .002). Heterogeneity appeared to be very low according to the *Q* value (*Q*_(5)_ = 3.40; *p* = .64), *I* ^2^ metric (*I* ^2^ = 0.00%; 95% CI, 0%, 86.32%) and PI (95% PI, −1.21, −0.46). The same six comparisons (five studies) that included depression effect sizes suggested a moderate effect at follow‐up (*g* = −0.40; 95% CI, −0.76, −0.05; *p* = .031), with very low heterogeneity (*Q*_(5)_ = 3.17; *p* = .67; *I* ^2^ = 0%; *I* ^2^ 95% CI, 0%, 77.24%; 95% PI, −0.76, −0.05). In relation to worry, five comparisons (four studies; treatment: *n* = 112, control: *n* = 62) evaluated outcomes at follow‐up time points ranging from 1‐ to 6‐months. This revealed a nonsignificant moderate effect (*g* = −0.35; 95% CI, −0.86, 0.17; *p* = .13), with limited heterogeneity (*Q*_(4)_ = 4.95; *p* = .29; *I* ^2^ = 10.44%; *I* ^2^ 95% CI, 0%, 92.64%; 95% PI, −0.97, 0.28).

### 3.8 Sensitivity analysis

Sensitivity analyses were conducted to evaluate the potentially confounding effect of the inclusion of two studies in which not the full sample presented with a GAD diagnosis but rather a small percentage presented with significant subthreshold symptoms only. Random‐effect models conducted without the two studies in question (E. Andersson et al., 2017; Newby et al., 2013) suggested similar effect estimates across four of the constructs (anxiety: *g* = −0.77; 95% CI, −1.05, −0.50; *p* < .0001; worry: *g* = −0.70; 95% CI, −0.93, −0.48; *p* < .0001; depression: *g* = −0.67; 95% CI, −0.87, −0.47; *p* < .0001; functional impairment: *g* = −0.64; 95% CI, −0.88, −0.39; *p* < .0001). Only quality of life, excluding E. Andersson et al. (2017), resulted in a nonsignificant effect estimate (*g* = 0.29; 95% CI, −0.05, 0.64; *p* = .080); however, this may have been due to the limited number of studies remaining within this analysis (i.e., only five studies included measures of quality of life apart from E. Andersson et al., 2017). In addition, mixed‐effect models (using REML estimation and the Knapp–Hartung method) were used to evaluate the possibility that outcomes were dependent on which outcome measure was used to assess each construct within each study. Omnibus tests across these mixed‐effect models were nonsignificant, confirming that the outcome measure used within each study did not predict effects found across all of the five constructs (see Appendix B and Table B1 for details).

### 3.9 Moderator analyses

Several potential moderators of effects were explored across the primary outcomes of anxiety and worry using mixed‐effect models. Other study level moderators were deemed not feasible for inclusion due to the limited number of studies within each subgroup (type of control group, support during intervention, referral type). A trend towards significance was observed for intervention type (conventional CBT vs. other interventions) in relation to anxiety outcomes (*b* = −0.45; 95% CI, −0.95, 0.04; *p* = .068), accounting for 14.06% of heterogeneity among effect sizes then. Examination of effect estimates by subgroup suggested a larger effect among the 17 iCBT studies (*g* = −0.93; 95% CI, −1.20, −0.65; *p* < .0001) than the six studies which implemented other interventions (*g* = −0.47; 95% CI, −0.88, −0.06; *p* = .027). There was more heterogeneity present among studies in the latter group (iCBT: *Q*_(16)_ = 34.08; *p* = .005; *I* ^2^ = 55.07%; other interventions: *Q*_(5)_ = 17.75; *p* = .003; *I* ^2^ = 70.87%), probably due to its theoretical diverseness.

The length of treatment (in weeks) emerged as a significant moderator of effects for anxiety (*b* = −0.15; 95% CI, −0.24, −0.06; *p* = .003) and worry outcomes (*b* = −0.16; 95% CI, −0.26, −0.05; *p* = .005), accounting for 66.21% and 54.39% of heterogeneity among studies, respectively, and suggesting that longer treatment was associated with better outcomes. The remaining moderators (average age of sample, intervention scope, baseline severity, number of modules in intervention, average percentage of program completed at posttreatment, average minutes spend by supporters per participant) remained nonsignificant across anxiety and worry models. For details see Appendix B and Table B2. Average baseline symptom scores across studies are reported in Appendix B and Table B3

### 3.10 Publication bias

**Figure B6:**
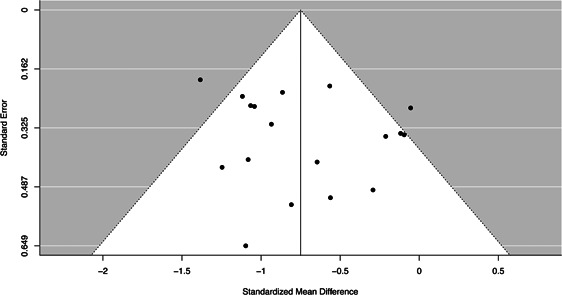
Funnel plot of studies included in worry random‐effect models

**Figure B7:**
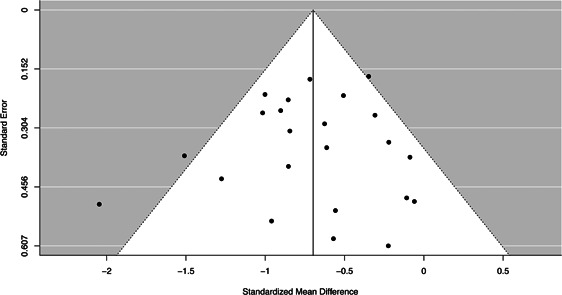
Funnel plot of studies included in depression random‐effect models

A funnel plot generated across studies assessing anxiety suggested some possible asymmetry; however, given the heterogeneity found within the random‐effect model, as described above, and Egger's test being nonsignificant (*t*_(21)_ = 0.06; *p* = .95), it was concluded that publication bias was unlikely to be substantial. For worry, asymmetry within the funnel plot seemed minimal and Egger's test was not significant (*t*_(16)_ = 0.73; *p* = .47). In terms of depression outcomes, the funnel plot flagged no asymmetry and this was confirmed by Egger's test (*t*_(21)_ = −0.24; *p* = .81). For funnel plots, see Appendix B, Figures B7, B1, B6. Due to the smaller number of studies contained in the functional impairment and quality of life analyses, it was not feasible to assess publication bias across those constructs.

## 4 DISCUSSION

The main aim of the current study was to evaluate the effectiveness of Internet‐delivered interventions in treating symptoms of GAD. We updated Richards et al. (2015) meta‐analysis to now include a total of 20 studies. Effect sizes for the primary outcomes of anxiety (*g* = 0.79) and worry (*g* = 0.75) were found to be large and in favor of Internet‐delivered interventions, confirming the value of this mode of treatment among individuals experiencing GAD. In addition, Internet‐delivered interventions also appeared to be effective in treating comorbid symptoms of depression, to improve overall levels of functioning and have at least some effect on quality of life. While controlled follow‐up evaluations of Internet‐delivered interventions continue to be limited, the current study provides some evidence that outcomes are maintained into follow‐up, at least in terms of symptoms of anxiety and depression.

These findings confirm and extend Richards et al.'s (2015) findings and are in line with recent meta‐analyses evaluating psychological treatments for GAD (Carl et al., 2020) and iCBT for depression and anxiety (Andrews et al., 2018). While both studies found comparable large effect sizes across their primary outcomes (*g* = 0.76, *n* = 39; *g* = 0.80, *n* = 64), crucially neither focused on the effects of Internet‐delivered interventions on GAD specifically, with the first also including non‐Internet‐delivered interventions and the latter only including Internet‐delivered interventions based on CBT. This differentiation is key, as the sole focus on the Internet as a treatment modality, irrespective of ever debated psychotherapeutic models, is what may help to fill existing gaps in the provision of treatment to those suffering from GAD (Alonso et al., 2018; Richards et al., 2020). In terms of secondary findings, beneficial large effects of psychological interventions for GAD on depressive symptoms have also been previously reported (Carl et al., 2020; Cuijpers et al., 2016) and are encouraging considering frequent comorbidity between MDD and GAD and close associations between anxiety and depressive symptoms (Jacobson & Newman, 2017; Moffitt et al., 2007). Similarly, our findings of large co‐occurring functional improvements are important in the context of close ties between anxiety, depression, and functioning, especially in the longer term (Iancu et al., 2014; Lukat et al., 2017).

Nevertheless, meta‐analytic results also suggested significant variability in effect sizes among included studies, which, at least in part, may need to be understood in the context of significant variability among the Internet‐delivered interventions implemented within these studies. Not only did interventions vary in how long participants had access to them, but more importantly they included various components and aimed to address various underlying mechanisms in line with the particular theoretical model they adhered to. Interestingly though and independent of those varying intervention characteristics, our analyses suggested that longer treatment durations are associated with better outcomes in terms of GAD symptoms. While more research is needed to understand the intricacies of dose–response relationships in Internet‐delivered intervention (Enrique et al., 2019; McVay et al., 2019), this finding may suggest that time, or at least some minimum amount of time, is of the essence in allowing various “active ingredients” of specific Internet‐delivered interventions to work. This would make sense in the context of established face‐to‐face treatments for GAD usually spanning 16+ weeks (American Psychological Association, 2016).

Internet‐delivered interventions drawing on a wealth of psychological theories have been developed and implemented. Still, conventional “second wave” CBT‐based interventions continue to dominate the field. As such, only comparisons between iCBT and other interventions (drawing on various theoretical models including “third wave” CBT) were feasible, which did suggest somewhat larger effects across conventional CBT‐based interventions. Importantly, interventions following other theoretical models were also found to be effective though, and in light of users not necessarily choosing iCBT when given the option and the effects not meeting user preferences can have (Lindegaard et al., 2020; Williams et al., 2016), the further development and implementation of intervention grounded in different paradigms seems advisable. Here, as more research becomes available it will be particularly interesting to explore the effectiveness of “second wave” CBT interventions in comparison to their “third wave” counterparts, as these have become increasingly popular in the treatment of GAD in recent years.

### 4.1 Limitations

The study has several limitations. First, due to the limited number of studies, we could not properly evaluate the influence of a number of moderators on our findings; among them, the effects of type of control group. While we could not assess this quantitatively, it seems effect sizes among studies utilizing active control groups may have been smaller and placebo effects may have played a role in large effects we found across waitlist‐controlled studies. Second, effect sizes at follow‐up were based on only a rather small number of studies that were controlled at follow‐up and we did not include any within‐group comparisons. Therefore, follow‐up evidence can be only treated as preliminary at this time. Third, the number of studies we included in our analyses was insufficient to conduct more advanced meta‐analytic procedures like multilevel meta‐analysis or robust variance estimation, better equipped to model the complexity of the data we extracted. Fourth, in examining multiple moderators across two constructs (anxiety and worry), type I error rates might have been inflated, making false positive findings more likely.

## 5 CONCLUSION

Internet‐delivered interventions are a viable option for the treatment of GAD and ought to be considered by clinicians and policy makers alike in the interest of addressing existing gaps in the provision of treatment of GAD. Since 2015, the field of Internet‐delivered interventions has matured and the current meta‐analysis was able to utilize data from 20 reasonably high quality studies, with the body of evidence as a whole appearing unlikely to have been unduly biased by methodological weaknesses at this point. Future research should investigate sources of heterogeneity in intervention effects to further develop, improve, and disseminate Internet‐delivered interventions.

## CONFLICT OF INTERESTS

Nora Eilert, Angel Enrique, Rebecca Wogan, Olwyn Mooney, and Derek Richards are employees of SilverCloud Health, developer of computerized psychological interventions for depression, anxiety, stress, and comorbid long‐term conditions. Timulak serves as a research consultant for SilverCloud Health. One study included in the meta‐analysis was authored by Derek Richards, Angel Enrique, Nora Eilert, and Ladislav Timulak among others and part funded by SilverCloud Health.

## Figures and Tables

**Figure B5:**
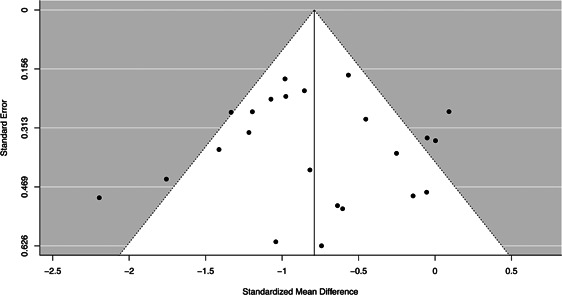
Funnel plot of studies included in anxiety random‐effect models

## Data Availability

Data analyzed within this study are available upon request to the corresponding author.

## APPENDIX A

**PRISMA checklist**

**Table:**

Section/topic	#	Checklist item	Reported on page #
**TITLE**			
Title	1	Identify the report as a systematic review, meta‐analysis, or both	1
**ABSTRACT**			
Structured summary	2	Provide a structured summary including, as applicable: background; objectives; data sources; study eligibility criteria, participants, and interventions; study appraisal and synthesis methods; results; limitations; conclusions and implications of key findings; systematic review registration number	2
**INTRODUCTION**			
Rationale	3	Describe the rationale for the review in the context of what is already known	4–5
Objectives	4	Provide an explicit statement of questions being addressed with reference to participants, interventions, comparisons, outcomes, and study design (PICOS)	5
**METHODS**			
Protocol and registration	5	Indicate if a review protocol exists, if and where it can be accessed (e.g., web address), and, if available, provide registration information including registration number	1, 5, 36
Eligibility criteria	6	Specify study characteristics (e.g., PICOS, length of follow‐up) and report characteristics (e.g., years considered, language, publication status) used as criteria for eligibility, giving rationale	6–7
Information sources	7	Describe all information sources (e.g., databases with dates of coverage, contact with study authors to identify additional studies) in the search and date last searched	6
Search	8	Present full electronic search strategy for at least one database, including any limits used, such that it could be repeated	6
Study selection	9	State the process for selecting studies (i.e., screening, eligibility, included in systematic review, and, if applicable, included in the meta‐analysis)	6–7
Data collection process	10	Describe method of data extraction from reports (e.g., piloted forms, independently, in duplicate) and any processes for obtaining and confirming data from investigators	7–8
Data items	11	List and define all variables for which data were sought (e.g., PICOS, funding sources) and any assumptions and simplifications made	7–10, 18, 31
Risk of bias in individual studies	12	Describe methods used for assessing risk of bias of individual studies (including specification of whether this was done at the study or outcome level), and how this information is to be used in any data synthesis	8–9
Summary measures	13	State the principal summary measures (e.g., risk ratio, difference in means)	9
Synthesis of results	14	Describe the methods of handling data and combining results of studies, if done, including measures of consistency (e.g., *I* ^2^) for each meta‐analysis	9
Risk of bias across studies	15	Specify any assessment of risk of bias that may affect the cumulative evidence (e.g., publication bias, selective reporting within studies)	8–9
Additional analyses	16	Describe methods of additional analyses (e.g., sensitivity or subgroup analyses, meta‐regression), if done, indicating which were prespecified	10
**RESULTS**			
Study selection	17	Give numbers of studies screened, assessed for eligibility, and included in the review, with reasons for exclusions at each stage, ideally with a flow diagram	11–12
Study characteristics	18	For each study, present characteristics for which data were extracted (e.g., study size, PICOS, follow‐up period) and provide the citations	12–18
Risk of bias within studies	19	Present data on risk of bias of each study and, if available, any outcome‐level assessment (see Item 12)	20‐21
Results of individual studies	20	For all outcomes considered (benefits or harms), present, for each study: (a) simple summary data for each intervention group and (b) effect estimates and confidence intervals, ideally with a forest plot	18–20; Appendix B
Synthesis of results	21	Present results of each meta‐analysis done, including confidence intervals and measures of consistency	21–24 Appendix B
Risk of bias across studies	22	Present results of any assessment of risk of bias across studies (see Item 15)	20–21
Additional analysis	23	Give results of additional analyses, if done (e.g., sensitivity or subgroup analyses, meta‐regression [see Item 16])	25–27
**DISCUSSION**			
Summary of evidence	24	Summarize the main findings including the strength of evidence for each main outcome; consider their relevance to key groups (e.g., healthcare providers, users, and policy makers)	27–28, 30
Limitations	25	Discuss limitations at study and outcome level (e.g., risk of bias), and at review level (e.g., incomplete retrieval of identified research, reporting bias)	29
Conclusions	26	Provide a general interpretation of the results in the context of other evidence, and implications for future research	28–30
**FUNDING**			
Funding	27	Describe sources of funding for the systematic review and other support (e.g., supply of data); role of funders for the systematic review	31

## APPENDIX B

Additional results:

**Table B3:** Baseline symptom levels across study interventions

Study (year)	Baseline mean scores (*SD*)					
	Anxiety		Depression		Worry	
G. Andersson et al. (2012)	GAD‐IV	10.63 (1.15)	BDI‐II	18.11 (7.89)	PSWQ	68.21 (6.22)
	GAD‐IV	10.89 (1.17)	BDI‐II	17.82 (6.93)	PSWQ	69.13 (5.9)
E. Andersson et al. (2017)	HAD‐A	11.41 (3.91)	MADRS‐S	16.02 (6.54)	PSWQ	66.1 (6.52)
Bell et al. (2012)	GADI	31.05 (9.1)	BDI‐II	21.45 (13.2)	PSWQ	66.65 (9.25)
Berger et al. (2014)	BAI	34.44 (7.63)	BDI‐II	22.26 (9.64)	PSWQ	66.37 (8.35)
	BAI	34.41 (7.87)	BDI‐II	24.53 (9.8)	PSWQ	65.04 (6.24)
Christensen et al. (2014)	GAD‐7	11.6 (4.25)	CES‐D	25.25 (9.7)	‐	‐
Dahlin et al. (2016)	GAD‐7	13.67 (3.9)	PHQ‐9	11.29 (4.78)	PSWQ	67.17 (6.97)
Dear et al. (2015)	GAD‐7	12.26 (4.83)	PHQ‐9	10.86 (4.37)	‐	‐
Hirsch et al. (2018)	GAD‐7	14.28 (3.32)	PHQ‐9	13.14 (4.19)	PSWQ	68.29 (6.38)
Johansson et al. (2013)	GAD‐7	12.67 (3.18)	PHQ‐9	13.3 (3.74)	‐	‐
L. Johnston et al. (2011)	GAD‐7	13.84 (3.77)	PHQ‐9	12.8 (5.97)	PSWQ	68.25 (7.89)
	GAD‐7	13.76 (4.28)	PHQ‐9	13.27 (5.64)	PSWQ	65.47 (11.44)
Jones et al. (2016)	GAD‐7	14.55 (3.16)	PHQ‐9	15.68 (5.32)	PSWQ‐A	31.49 (6.56)
Mullin et al. (2015)	GAD‐7	9.03 (4.62)	PHQ‐9	10.87 (5.4)	‐	‐
Newby et al. (2013)	GAD‐7	10.4 (4.37)	PHQ‐9	11.01 (4.35)	PSWQ	63.67 (10.22)
Paxling et al. (2011)	GAD‐IV	10.21 (2.28)	MADRS‐S	17.3 (8.86)	PSWQ	69.03 (6.25)
Richards et al. (2020)	GAD‐7	13.84 (4.18)	PHQ‐9	14.77 (5.18)	‐	‐
Robinson et al. (2010)	GAD‐7	12.42 (3.73)	PHQ‐9	12.29 (4.97)	PSWQ	64.47 (9.85)
	GAD‐7	12.7 (4.11)	PHQ‐9	11.95 (4.68)	PSWQ	64.92 (9.76)
Salemink et al. (2014)	STAI‐T	59.35 (8.3)	BDI	24.04 (10.71)	‐	‐
Titov et al. (2010)	GAD‐7	‐	PHQ‐9	‐	PSWQ	65.68 (9.28)
Titov et al. (2011)	GAD‐7	11.40 (3.76)	PHQ‐9	13.04 (6.57)	PSWQ	59.89 (8.26)
Titov et al. (2009)	GAD‐7	13.98 (4.01)	PHQ‐9	12.29 (5.72)	PSWQ	66.23 (10.48)

Abbreviations: BAI, Beck Anxiety Inventory; Beck Depression Inventory‐II; CES‐D, Centre for Epidemiological Studies Depression Scale; GAD‐IV, Generalized Anxiety Questionnaire‐IV; GAD‐7, Generalized Anxiety Questionnaire‐7; HAD‐A, Hospital Anxiety and Depression Scale‐Anxiety; HAD‐D, Hospital Anxiety and Depression Scale‐Depression; MADRS‐S, Montgomery–Asberg Depression Scale; PHQ‐9, Patient Health Questionnaire–9 item; PSWQ, Penn State Worry Questionnaire; PSQW‐A, Penn State Worry Questionnaire‐Abbreviated; STAI‐T, State‐Trait Anxiety Inventory‐Trait; WSAS, Work and Social Adjustment Scale.

**Table B1:** Omnibus test of the effect of using different outcome measures to assess each construct

	*F*	*df*1, *df*2	*p*
Anxiety	0.67	5, 17	.65
Worry^a^	n/a	n/a	n/a
Depression	0.31	3,19	.82
Impaired functioning	0.13	2, 7	.88
Quality of life	3.60	3, 2	.16

^a^ Seventeen studies used the Penn State Worry Questionnaire, one used the Penn State Worry Questionnaire‐Abbreviated.

**Table B2:** Results for moderators explored across anxiety and worry outcomes

	Anxiety			Worry		
Moderator	*b*	95% CI	*p*	*b*	95% CI	*p*
Average age of sample	−0.02	−0.05, 0.00	.083	−0.00	−0.04, 0.03	.75
Intervention scope (disorder‐specific vs. transdiagnostic)	0.05	−0.45, 0.56	.82	0.02	−0.47, 0.44	.93
Intervention type (iCBT vs. other)	−0.45	−0.95, 0.04	.068	−0.10	−0.60, 0.4	.67
Relative baseline symptom severity^a^	0.07	−0.29, 0.15	.51	0.01	−0.10, 0.13	.81
Length of treatment (in weeks)	−0.15	−0.24, −0.06	.003	−0.16	−0.26, −0.05	.005
Number of modules in intervention	0.12	−0.05, 0.29	.16	0.09	−0.09, 0.26	.30
Average percentage of intervention completed at posttreatment^b^	−0.35	−0.92, 0.23	.22	0.13	−0.36, 0.63	.57
Average minutes spend by supporters per participants during treatment^c^	0.00	−0.01, 0.01	.80	−0.00	−0.01, 0.00	.35

Abbreviations: CI, confidence interval; GAD, generalized anxiety disorder; iCBT, Internet‐delivered cognitive behavioral therapy; PSWQ, Penn State Worry Questionnaire.

^a^ In terms of baseline symptom severity, moderator analyses were only feasible across two outcome measures (GAD‐7: *n* = 15 and PSWQ: *n* = 17), with the numbers of studies reporting on the remaining outcome measures being too small to facilitate mixed‐effect models.

^b^ The average percentage of intervention completed at posttreatment variables was coded in line with four categories (1, 0%–25%; 2, 26%–50%; 3, 51%–75%; 4, 76%–100%). All but one study (Richards et al., 2020) fell into Categories 3 and 4, thus only those categories were included in mixed‐effect models.

^c^ Mixed‐effect models included 15 studies for anxiety and 13 for worry.
